# Effect of Methyl Jasmonate on Thymol, Carvacrol, Phytochemical Accumulation, and Expression of Key Genes Involved in Thymol/Carvacrol Biosynthetic Pathway in Some Iranian Thyme Species

**DOI:** 10.3390/ijms222011124

**Published:** 2021-10-15

**Authors:** Farzad Kianersi, Alireza Pour-Aboughadareh, Mohammad Majdi, Peter Poczai

**Affiliations:** 1 Department of Agronomy and Plant Breeding, Faculty of Agriculture, Bu-Ali Sina University, Hamedan P.O. Box 6517838695, Iran; f.kianersi@agr.basu.ac.ir; 2 Seed and Plant Improvement Institute, Agricultural Research, Education and Extension Organization (AREEO), Karaj P.O. Box 3183964653, Iran; 3 Department of Agronomy and Plant Breeding, Faculty of Agriculture, University of Kurdistan, Sanandaj P.O. Box 1517566177, Iran; majdi60@gmail.com; 4 Research Center for Medicinal Plant Breeding and Development, University of Kurdistan, Sanandaj P.O. Box 1517566177, Iran; 5 Botany Unit, Finnish Museum of Natural History, University of Helsinki, P.O. Box 7, 00014 Helsinki, Finland

**Keywords:** *Thymus* species, phytochemical components, Methyl jasmonate, gene expression

## Abstract

Thyme species are a good source of thymol and carvacrol, which play a key role in controlling diseases. For the first time, the expression patterns of γ-terpinene synthase (TPS2), *CYP71D178*, and *CYP71D180* genes and the amount of phenolics compounds were evaluated in *T. migricus* and *T. daenensis* after different methyl jasmonate (MeJA) treatments. The highest thymol and carvacrol contents were observed in *T. migricus* (86.27%) and *T. daenensis* (17.87%) at MeJA 100 µM, which was consistent with the expression patterns of the three investigated genes. All species treated showed high total phenolic and flavonoid content compared to control plants for which the highest amounts were observed in *T. vulgaris* treated with 100 µM and 10 µM MeJA. Furthermore, in the 100 µM MeJA treatment, the relative expression of *TPS2* and *CYP71D178* in *T. migricus* increased 7.47 and 9.86-fold compared with the control, respectively. The highest level of *CYP71D180* transcripts (5.15-fold) was also observed for *T. daenensis* treated. This finding highlights the notion that thymol was known as the dominant component of the essential oil rather than carvacrol in diffident thyme species. This implies that MeJA at different concentrations influenced metabolic pathways and induced expression changes, resulting in a rise in essential oil levels.

## 1. Introduction

Thymus (*Thymus* spp.) is a perennial evergreen aromatic plant in the Lamiaceae family that is grown in many countries for its therapeutic properties, for example as an antioxidant, antimicrobial, or anti-inflammatory [1,2,3]. The major ingredients in thyme essential oils are thymol and carvacrol, which is a phenol monoterpenes derivative with pharmacological properties such as anti-metastatic activity, anti-oxidative, antimicrobial, and anti-inflammatory [4,5]. Due to the excellent quality of the essential oil (EO) derived from its aerial portions, certain Thymus species have been discovered for medicinal, food, and cosmetic uses [6].

*T. migricus* is a perennial plant with pinkish blooms that belongs to the section *Kotschyani* and is one of the endemic species cultivated in Iran. It is a well-known fragrant plant in northwest Iran, where it is frequently used for herbal tea and as a flavoring ingredient [7]. Members of the genus *Thymus* L. are recognized for their therapeutic qualities, which include antibacterial, antimicrobial, antiviral (*T. vulgaris* L.), and antifungal (*T. daenensis* Celak.) capabilities [2,5,6,8].

Thymus species such as *T. migricus* [4] and *T. daenensis* [5], similar to some other plants from the Lamiaceae family, have strong antioxidant capabilities due to their abundant source of phenolic compounds. The shikimate and phenylpropanoid pathways generate phenolic chemicals, which are secondary metabolites. These chemicals have strong antioxidant properties and might help prevent diabetes, cardiovascular disease, and neurological disorders [9,10].

Thyme essential oil has major medicinal applications and is widely utilized in the food and cosmetics industries as a flavoring ingredient. *Thymus vulgaris* essential oil is mostly made of monoterpene combinations. Thymol and its isomer carvacrol are the most common components of essential oils [11,12]. As a result of their antitussive, antioxidant, expectorant, antimicrobial, and antispasmodic characteristics, thymol and carvacrol have significant therapeutic significance [13,14].

**Figure 1:**
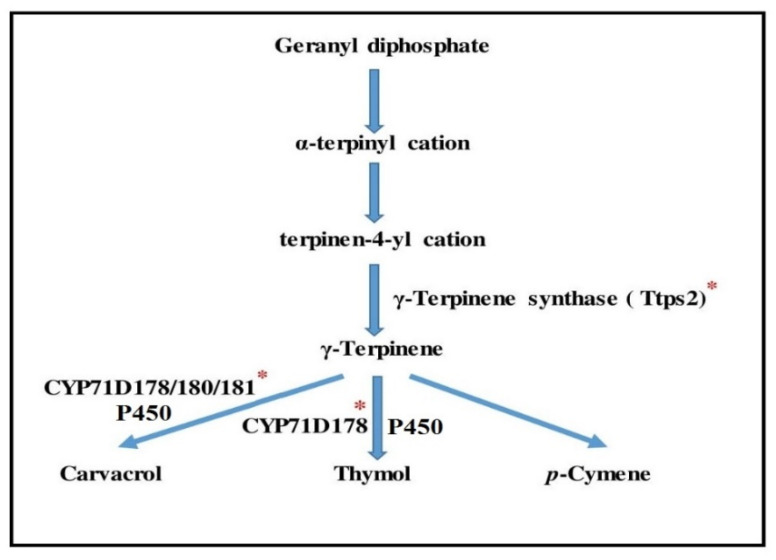
The thymol biosynthetic pathway. A red star is placed above enzyme genes that have been identified in thymus.

Thymol, one of the recognized chemicals in thyme, has received much press due to its potential to combat gum disease, tooth decay, infection, and foul breath. Carvacrol and thymol are discovered in the aerial portions of thyme, and the biosynthetic pathways for thymol and carvacrol have recently been discovered [15]. Dimethylallyl diphosphate (DMADP) and isopentenyl diphosphate (IDP), which are generated through the MEP (methyl erythritol phosphate) pathway, are used to biosynthesize thymol and carvacrol as phenol monoterpenes (Figure 1) [16]. 1-deoxylulose-5-phosphate (DXP) is transformed irreversibly to 2C-methylderithritol-4-phosphate (MEP) in the MEP pathway by 1-deoxyDxylulose-5-phosphate reductoisomerase (DXR). This step is the first and most important step in the MEP route [17]. In this metabolic route, geranyl diphosphate synthase (GDS) is an important enzyme. It can catalyze the condensation of IDP and DMADP into geranyl diphosphate (GDP), which is a universal monoterpene precursor [18,19]. Following that, γ-terpinene synthase (TPS), a monoterpene synthase, generates γ-terpinene by cyclizing GDP. Furthermore, cytochrome P450 (CYP) monooxygenases *CYP71D178*, *CYP71D180*, and *CYP71D181* are involved in further alteration of the γ-terpinene skeleton to generate thymol and carvacrol, so that only *CYP71D178* produces thymol, while *CYP71D178*, *CYP71D180*, and *CYP71D181* biosynthesize carvacrol (Figure 1).

**Figure 2:**
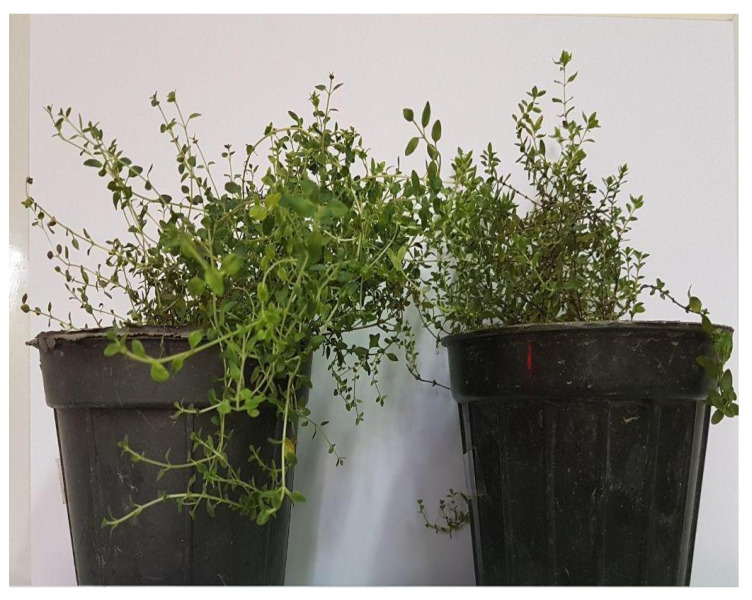
Treated leaf of Thyme plants with different treatments of MeJA under greenhouse condition.

Although the expression of these genes has been investigated in several plant species, especially in *T.* vulgar [20], the thymol/carvacrol biosynthetic pathway genes in Iranian thyme (Figure 2), *T. migricus* and *T. daenensis*, are yet to be investigated.

In recent years, a slew of studies have looked into the effects of biological and non-biological inducers of the biosynthesis of secondary metabolites such as ultraviolet (UV) irradiation [21], trans-cinnamic acid [22], methyl jasmonate (MeJA), and salicylic acid (SA) [23,24,25,26,27]. In plants, jasmonic acid (JA) and its methyl ester, methyl jasmonate, have been shown to have a function in signal transduction and may control defense genes. It is also extensively utilized exogenously in plants to stimulate secondary metabolite synthesis [23,28].

Methyl jasmonate, as elicitor phytohormones, are another family of abiotic stressors that cause a cascade of physiological reactions that alter plant morphology and result in secondary metabolite production and accumulation [9,29]. Therefore, phytohormones can be intentionally given to plants to boost their phytochemical content. The chemical composition of essential oils and extracts in plant species is generally affected by these variables. According to the research, MeJA and SA therapy stimulates secondary metabolism in many plant species, allowing plant hormones to control the synthesis of volatile chemicals, which is linked to plant protection and interactions with the environment [30,31,32].

Although thyme is a valuable therapeutic herb, our review of the literature revealed that no studies have been performed to date regarding the effects of MeJA on phytochemical properties and thymol/carvacrol biosynthesis pathway gene expression in Iranian thymus. Therefore, the overall phenolic and flavonoid contents, as well as the concentration of thymol and carvacrol, were determined in this study. Finally, following MeJA treatment, the gene expression patterns of genes implicated in the thymol and carvacrol biosynthesis pathways, such as γ-terpinene synthase (TPS2), *CYP71D178*, and *CYP71D180*, were examined in thymus species, especially in two Iranian species, for the first time.

As a result, we sought to determine the concentrations of thymol and carvacrol, which are two important compounds found in thymus leaves after the plant was elicited with MeJA. We also attempted to establish a relationship between the pattern of expression of certain thyme biosynthesis pathway genes and the changed content of thyme in the leaves of thymus plants at the vegetative development stage after treating them with MeJA.

## 2. Result and Discussion

### 2.1. Effect of Different Concentrations of MeJA on Thymol and Carvacrol Content in Fennel Leaves

**Figure 3:**
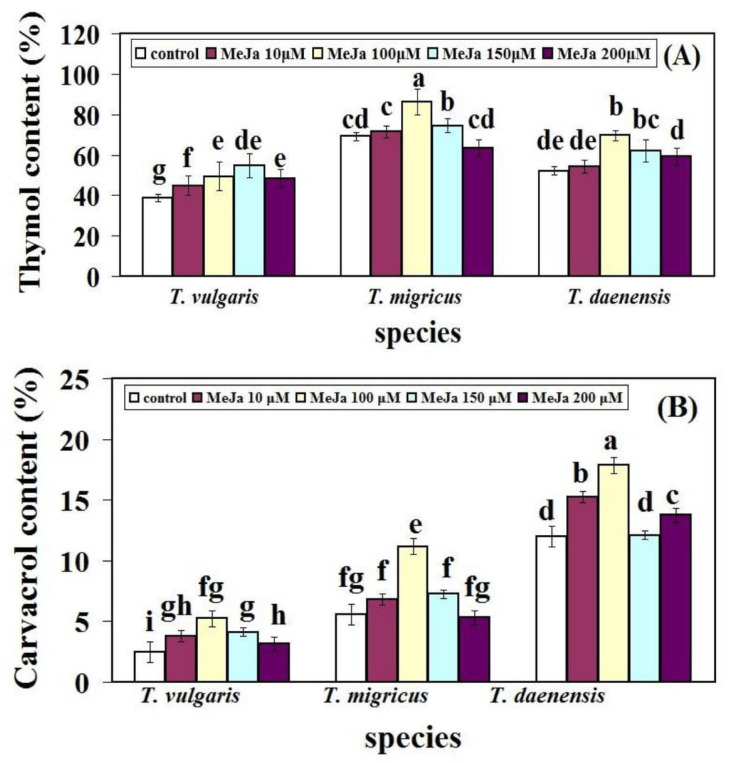
Thymol (**A**) and carvacrol (**B**) content of leaves of thymus species treated with different treatments of MeJA. Bars with different letters mean significant at 1% level of probability according to Duncan’s test.

In the present study, monoterpenes thymol and carvacrol were the major components of oil in three thymus species. MeJA treatments led to significant changes in thymol and carvacrol contents in studied species grown under greenhouse conditions (Figure 3). It was found that thymol and carvacrol contents were boosted with increasing concentrations of MeJA treatments when compared to the control condition. In *T. vulgaris,* the highest amount of thymol was observed at 150 µM MeJA (54.71%), which was approximately 1.41 times that of control leaves. The maximum thymol content was at 100 µM MeJA (86.27%), whereas the minimum one was at 200 µM MeJA (63.33%) in *T. migricus.* In addition, the highest thymol content (69.67%) was in *T. daenensis* treated with 100 µM MeJA, compared to the control (Figure 3A).

In the case of carvacrol content, 100 µM MeJA showed the highest production (5.25%) in *T. vulgaris*. The highest detected amount of carvacrol in *T. migricus* was observed at 100 µM MeJA treatment (11.16%), which was approximately two-fold higher than the content in the control. The lowest amount was observed at 200 µM MeJA (5.32%) (Figure 3B).

Our results revealed an enhancement of carvacrol production during 24 h of treatment until 100 µM MeJA elicitors. MeJA treatment, at 150 µM, showed no significant change in the carvacrol content compared to the control. However, at 100 µM MeJA, carvacrol content increased to 17.87% in *T. daenensis* (Figure 3B).

A comparison of carvacrol and thymol content in three thymus species grown under different MeJA concentrations revealed that the highest thymol and carvacrol contents were related to *T. migricus* and *T. daenensis,* respectively, at 100 µM MeJA treatment. This concentration had the best effect on thymol and carvacrol contents in each species except *T. vulgaris* compared to control (Figure 3). It was assumed that thymol was considered as the major constituent of the oil rather than carvacrol due to its percentage. This finding proves the dominance of thymol in diffident thyme species. The increase in thymol and carvacrol contents as a result of MeJA might be due to the stimulation of metabolic pathways and overexpression of related genes such as cryptochromes to activate radical scavenging by phenolic compounds. Tohidi et al. [33] found similar variations in thymol concentration between *T. migricus* and other species they investigated. However, in field circumstances, these authors found a considerably greater thymol content, which is most likely due to environmental stressors. Mohammadi et al. [1] investigated the impact of various environmental variables, such as drought, on increased amounts of several key components such as thymol, carvacrol, and linalool in *T. kotschyanus* and *T. vulgaris*, and they reported consistent findings. *T. vulgaris* and *Origanum vulgare* L. are two Lamiaceae species whose essential oil content and, as a result, thymol and carvacrol production and accumulation vary in various tissues or in response to distinct environmental variables and stressors [31,34].

### 2.2. Differences in Total Phenolic Content and Total Flavonoid Content in Response to Different MeJA Concentrations

**Figure 4:**
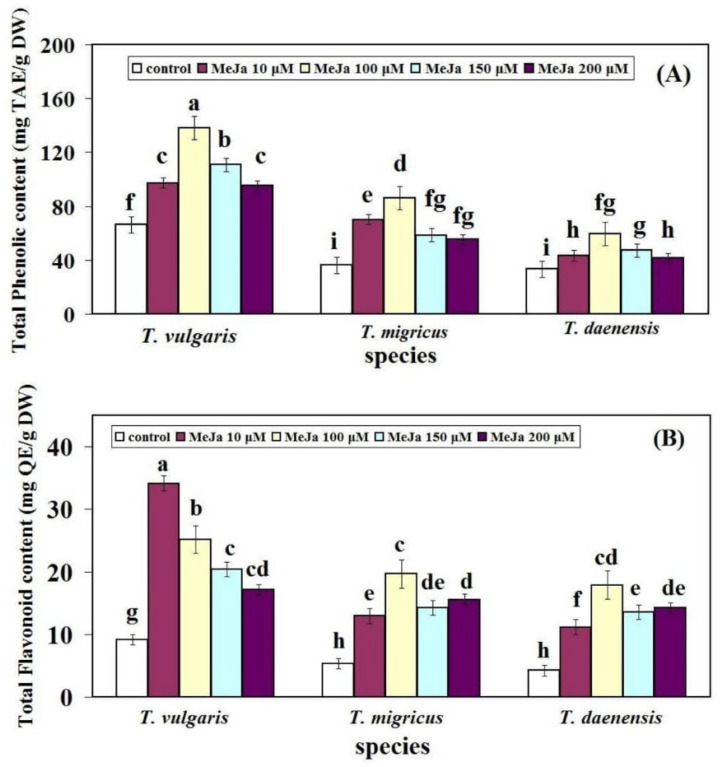
Influence of different treatments of MeJA on (**A**) TPC, and (**B**) TFC of thymus species. Bars with different letters mean significant at 1% level of probability according to Duncan’s test.

Total phenolic content (TPC) and total flavonoid content (TFC) are two commonly used markers that indicate total antioxidant activity in samples. Significant variations in TPC and TFC values were found in the Thymus samples tested in this investigation (Figure 4). All three species followed the same pattern in TPC value, with an increasing amount up to 100 µM in *T. vulgaris* (138.12 mg TAE/g DW), *T. migricus* (86.28 mg TAE/g DW), and *T. daenensis* (59.74 mg TAE/g DW) and then reducing until 200 µM MeJA (Figure 4A). *T. vulgaris* have the highest TPC at 100 µM MeJA among all species.

The Thymus samples investigated exhibited a high TFC value, with the highest found in *T. vulgaris* at 10 µM MeJA (34.14 mg QE/g DW), whereas in *T. migricus* and *T. daenensis*, it was found at 100 µM MeJA (19.68 and 17.88 mg QE/g DW, respectively) (Figure 4B).

These findings suggest that TPC and TFC levels in thyme plants vary depending on genotype and dose, which is in line with the findings of other researchers [35]. According to reported research, the Thymus species may be regarded as not only rich sources of phenolics and flavonoids but also potential sources of natural antioxidants.

In addition, several studies have attempted to increase the total flavonoids or phenolics in various plant species by applying MeJA externally to increase antioxidant, anti-proliferative, and antiadipogenic activity [36].

Furthermore, in all species of thyme for controls and all MeJA treatments, TPC values were higher than TFC, which is consistent with previous research [37]. Furthermore, the flavonoid content of plants can be affected by the application of certain solvents.

Previous study has shown that TPC levels vary based on the Thymus species studied [38]. Other species examined had shown similar variations in thymol concentration to *T. migricus*, and these differences have been reported before [39].

In *T. vulgaris*, Hossain et al. [40] discovered that methanol generated more flavonoids when compared to four other solvents. The scavenging process is the mechanism that underpins flavonoid activities. The region where the sample is taken, as well as the main meteorological and environmental variables, have a significant impact on the makeup of these chemicals [41].

It was reported that total phenols, flavonoids, and acacetin, a flavonoid molecule with various medicinal properties, were all increased by MeJa. In cell suspension extracts of *Scrophularia kakudensis* Franch, greater concentrations of MeJa substantially regulated the activities of antioxidant enzymes and increased the scavenging potentials of free radicals [42]. Furthermore, phenolic compounds can scavenge reactive oxygen intermediates without triggering further oxidative processes [43]. A previous study has revealed that TPC takes on different levels based on the Thymus species [38]. TPC values for *T. caramanicus* were determined to be greater by Safaei-Ghomi et al. [44] than by Tohidi et al. [39]. *T. spathulifolius* [45] and *T. Serpyllum* [46] have also been shown to exhibit high TPC values.

### 2.3. Induction of Essential Oil Yield (EO) by Using MeJa Elicitor

**Figure 5:**
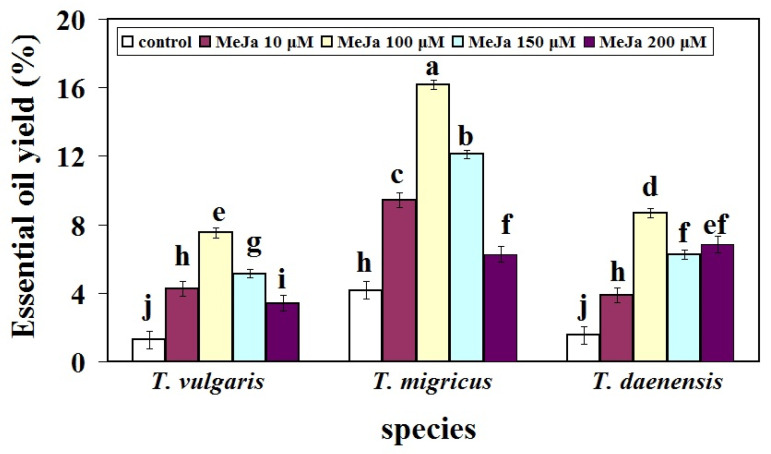
Thymus species essential oil yield changes under different treatments of MeJa. Bars with different letters mean significant at 1% level of probability according to Duncan’s test.

The results showed that there were significant differences and high variation for EO yield among three species at different MeJA concentrations (Figure 5), indicating that MeJA had a significant impact on EO yield. The EO yield was increased in all species up to 100 µM MeJA in comparison with control. *T. migricus* produced the maximum EO production at 100 μM MeJA (16.16%), whereas *T. vulgaris* produced the lowest percentage of EO at 200 μM MeJA (3.42%) (Figure 5).

In *T. vulgaris* and *T. daenensis* treated with MeJA 100 µM, EO levels were 7.53% and 8.69%, respectively, which were 6.1 and 5.89-fold more than control, respectively. This finding highlights the notion that, as shown in Figure 3A, the maximum EO production may be connected to the thymol concentration. This implies that MeJA may have influenced metabolic pathways, resulting in a rise in EO levels.

Environmental and genetic variables may be directly responsible for the increase in EO yield seen with MeJA treatment compared to those observed under control. These findings support the hypothesis that in comparison to the other species tested, *T. migricus* has a considerably larger capacity for EO generation at all MeJA concentrations. Finally, additional variables such as climate, drying technique, collecting period, and extraction method can affect the yield of EO in Thymus [47,48]. Tohidi et al. [33] observed the same result in *T. migricus* under various light treatments. Treatments with elicitors that simulate abiotic stress are effective methods for studying biosynthetic pathway responses. In accordance with this, Verma et al. [34] showed that the developmental stage and elicitors have a significant effect on the essential oil content and composition of the closely related species *Origanum vulgare*. Moreover, it has been reported that elicitors had considerable effects on the essential oil content and its composition in *Origanum vulgare* [35]. More recently, many research investigations have been carried out in order to investigate the effects of both biochemical and nonbiochemical elicitors on the production of secondary metabolites, such as methyl jasmonate and salicylic acid [23,24]. Most of the time, these variables alter the chemical composition of essential oils in different plant species. MeJA and SA treatments have been shown to stimulate secondary metabolism in a variety of plant species, and it is probable that the synthesis of volatile chemicals is controlled by these plant hormones, which are involved in plant defense and interaction with the environment [24,31].

### 2.4. Changes in Gene Expression in the Thymol/Carvacrol Biosynthesis Pathway with Different Concentration of MeJA

**Figure 6:**
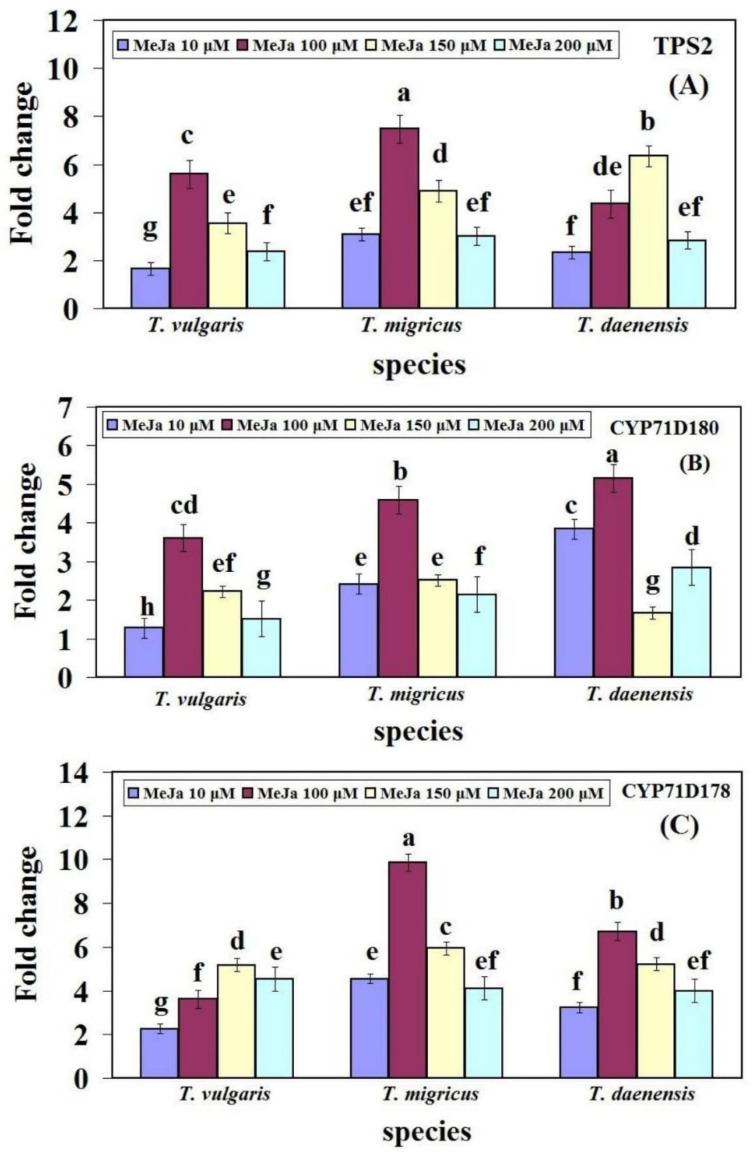
Relative expression of genes involved in thymol biosynthesis in thymus species leaves treated with different treatments of MeJA (**A**–**C**). Different letters indicate significance at 1% level of probability according to Duncan’s test.

The relative gene expression of *TPS2*, *CYP71D178*, and *CYP71D180* was investigated to examine how the thymol/carvacrol biosynthesis genes might be altered, 24 h after MeJA treatment began in leaves of *T. vulgaris*, *T. migricus*, and *T. daenensis*. For all genes in three species of thymus, significant differences (*p* ≤ 0.01) were observed related to different concentrations of MeJA. Significant changes in gene mRNA levels (TPS2, *CYP71D178*, and *CYP71D180*) were observed in response to during MeJA treatments, but the range of gene expression was species dependent (Figure 6).

Maximum expression levels of *TPS2* were observed at 100 µM MeJA in both *T. vulgaris* and *T. migricus,* which were 5.59- and 7.47-fold higher than control, respectively. The minimum expression levels in both *T. vulgaris* (1.64-fold) and *T. migricus* (3.1-fold) were found in plants treated with 10 µM MeJA. There was no significant difference between 10 and 200 µM MeJA in *T. migricus.* The highest levels of *TPS2* transcripts for *T. daenensis* were observed at 150 µM MeJA (6.34 fold)*,* whereas the lowest levels were found at 10 µM MeJA (2.34 fold) (Figure 6A).

*CYP71D180* upregulation was assessed at 100 µM MeJA in all three species. It was 3.61-fold higher than the control in *T. vulgaris*, while downregulation was observed at 10 µM MeJA. Both *T. migricus* and *T. daenensis* were 4.59- and 5.15-fold higher compared to the control at 100 µM MeJA. The lowest transcript level in *T. migricus* was at MeJA 200 µM, and in *T. daenensis*, it was at MeJA 150 µM (Figure 6B).

At 150 µM MeJA, the transcript level of *CYP71D178* was 5.18-fold higher than the non-treated leaves (Figure 6C). Expression increased sharply in *T. migricus* treated with 100 µM MeJA, with levels up to 9.86-fold higher than in the control (Figure 6C). In *T. daenensis*, the highest levels of *CYP71D178* transcripts were observed in leaves treated with 100 µM MeJA, reaching levels 6.72-fold higher than those in the control, but the expression levels were slightly lower (3.24 fold) at 10 µM MeJA in comparison to control (Figure 6C). Therefore at 100 µM MeJA, expression levels were highest for *TPS2* and *CYP71D178* in *T. migricus* and for *CYP71D180* in *T. daenensis.* These results demonstrated that 100 µM MeJA can be considered the optimum level of treatment to induce expression changes and stimulate the phytochemical compounds. In other words, our findings indicate that MeJA treatments may significantly increase the amount of thymol/carvacrol present in different species of thymus, and that the gene expression patterns of thymol/carvacrol biosynthesis-related genes are influenced by these elicitors.

As a result, MeJA treatment increases the production of secondary metabolites, especially thymol and carvacrol, to overcome oxidative stress. All examined genes in the thymol/carvacrol biosynthesis pathway reacted to MeJA, according to our findings. It increased transcription levels, although the magnitude of the rise varied depending on the species and gene of interest (Figure 6). According to the fold change, *CYP71D178* had the greatest rise in response to MeJA in all three species (Figure 6). The results show that *CYP71D178* is more impacted than *TPS2* and *CYP71D180*, indicating that it plays a critical role in thymol and carvacrol biosynthesis, which might explain the greater sensitivity to oxidative stress and increased expression levels.

The rise in thymol percentage found in the samples tested, rather than the increase in carvacrol content, suggests a biosynthetic relationship between the two substances [49]. For more information, Crocoll et al. (2010) hypothesized that the synthesis of thymol and carvacrol occurs directly from the c-terpinene substrate, and that p-cymene is produced as a by-product of the premature release of the substrate from the active site [50]. The increased expression of these genes may be due to an increase in the production of phenolic monoterpenes such as thymol and carvacrol, which are associated with these genes. An increase in the production and accumulation of phenolic monoterpenes has been reported to be induced by the presence or absence of ants in the vicinity of *Origanum vulgare* L., and this has been linked to the presence or absence of ants [31]. According to a recent study, the parasitization of *Origanum vulgare* by a Myrmica ant increased the expression of genes involved in carvacrol and thymol biosynthesis, such as *OvTPS2, CYP71D180, CYP71D179/182*, and *CYP71D178*, among others [31]. The same result was observed in the research of Majdi et al. [20], who studied the relative expression of these genes influenced by MeJA, SA, and UV-C treatments in *T. vulgaris*. Moreover, *TPS2*, *CYP71D178*, and *CYP71D180* genes in *T. migricus* have extremely comparable expression patterns in response to MeJA, suggesting that they are most likely regulated by the same or similar transcription factors. This has been reported previously for these elicitors and their effects on terpene biosynthesis in different plant species, such as *Picea abies* L. [51], *Vitis vinifera* L. [52], *Tanacetum parthenium* (L.) Sch. Bip. [23], and *Nigella sativa* L. [24].

As a result of a rise in phenolic monoterpenes such as thymol and carvacrol, it is possible that the enhanced expression of these genes in response to MeJA is a result of this increase. The presence or absence of ants in the vicinity of *Origanum vulgare* L. has recently been demonstrated to induce increased biosynthesis and the accumulation of phenolic monoterpenes [31], as well as in response to MeJA, SA, and UV-C treatments in *Tribulus vulgaris* [20]. According to our results, elicitation may enhance the synthesis of pharmaceutically active compounds in *T. vulgaris*, *T. migricus*, and *T. daenensis* without causing physical damage to the terpene biosynthesis or accumulation structures [51].

Using a combination of metabolomics and transcriptomics, researchers may be able to determine how secondary metabolites are regulated [53]. Terpene biosynthesis in *T. vulgaris*, *T. migricus*, and *T. daenensis*, as well as other species, may be achieved using this method. Many environmental variables influence secondary metabolite production in plants. Thus, the genetic foundation of responsiveness to the MeJA signal should have evolved in thymus species. Methyl jasmonate treatment alters the gene expression of many transcription factors as well as protein and metabolic levels [54]. Terpenoids are the most common and structurally varied category of plant secondary metabolites [55], according to our findings. Our research demonstrates that thymus species reacted quickly to the MeJA signal.

## 3. Materials and Methods

### 3.1. Plant Material and Growth

The *T. migricus*, *T. daenensis*, and *T. vulgaris* seeds, different species of *Thymus*, were supplied from the Research Institute of Forests and Rangeland, Tehran, Iran and authenticated using Flora Iranica [56]. After surface sterilization, seeds were planted in plastic pots containing a mixture soil texture of perlite and compost to be grown under controlled environment conditions (16 h day at 20–22 °C and 8 h night at 18–20 °C).

### 3.2. Elicitation of Thymus by Different Concentration of MeJa

Different concentrations of MeJA solutions (10, 100, 150, and 200 μM) and distilled water (control) were sprayed on the leaves of plants until runoff, and sampling was conducted 24 h after spraying each plant. Then, treated and untreated plants were frozen in liquid nitrogen and preserved at −80 °C until used for further analysis.

### 3.3. RNA Extraction and cDNA Synthesis

The total RNA of leaves of thymus species were isolated by RNAXTM_PLUS Kit (SinaClon Bioscience Co., Karaj, Iran) according to manufacturer’s instructions and cDNA synthesized using M-MuLV reverse transcriptase based on the method, as reported by previously [24].

### 3.4. qRT-PCR and Gene Expression Analysis

To investigate the effects of different concentrations of MeJA on the thymol biosynthetic pathway genes, analysis of gene expression (fold change) was carried out with 3 biological replicates and 3 technical replications of each replicate. The specific primers for qRT-PCR were designed according to the previously obtained conserved sequencing results of thymus species [50,57], and ubiquitin was used as a reference gene for normalization. The qRT-PCR conditions were set up as follows: initial denaturation step at 94 °C for 40 s followed by 40 cycles of 94 °C for 5 s (denaturation), 52–62 °C for 35 s (annealing), and a final elongation step at 72 °C for 25 s. The annealing temperatures were 54 °C for ubiquitin primer, 62 °C for TPS2, and 60 °C for *CYP71D178* and *CYP71D180* primers (Table 1). Normalized raw Ct values were used to compare the results of control and treated samples using the 2^−ΔΔCt^ (Fold change) method, as described previously [58].

### 3.5. Estimation of Total Phenolic Content and Total Flavonoid Content

To measure the total phenolic content (TPC), plant methanolic extract was obtained using 500 mg of the dried and ground leaf samples with 80% methanol (20 mL) in a shaker (120 rpm) at room temperature for one day. Then, the extract was filtered, and the total phenolic content (TPC) was quantified with the Folin–Ciocalteu reagent, as described previously [39]. Finally, after heating at 45 °C for 15 min, absorbance was measured at 765 nm, and the total phenolic content was calculated as mg tannic acid equivalent/g dry weight.

In addition, the total flavonoid content (TFC) was determined based on the aluminum chloride colorimetric method [59] with minor modifications. Briefly, 0.25 mg of extract was mixed with 1.25 mL of water and 0.75 μL of sodium nitrate, and it was kept in the dark for six minutes. Next, 0.15 μL of aluminum chloride (10%) was added to this mixture, and it was incubated in the dark for 300 s for the reaction to complete. Finally, 0.275 mL of water and 0.5 mL of sodium hydroxide solution (5%) were added and mixed. After reading the adsorption of the reaction solution at 510 nm, the total flavonoid content was expressed as mg quercetin equivalent/g dry weight.

### 3.6. Essential Oil Extraction

The isolation of essential oil was achieved by hydrodistillation of dried leaves of thymus, and then, essential oil was extracted using a Clevenger-type apparatus for 5 h. Essential oil yield (%) was measured using dry material based on the following formula [59]:

Essential oil (EO) (%) = [mass of EO obtained (gr)/mass of dry matter (gr)] × 100.

### 3.7. Quantification of Thymol and Carvacrol

GC-FID and GC/MS methods were used for the identification and quantification of main chemical composition (thymol and carvacrol) of studied *Thymus* essential oils. The analyses were performed with an Agilent 7890 gas chromatograph (Agilent Technologies, Palo Alto, CA, USA) equipped with a flame ionization detector (FID) fitted with a fused silica HP-5MS capillary column (30 m × 0.25 mm, 0.25 μm film thickness). The following operation conditions were performed at programmed temperature: (a) 3 min at 60 °C, (b) raising temperature from 60 to 120 °C at a rate of 3.0 °C min^−1^, and (c) raising temperature from 120 to 300 °C at a rate of 15 °C min^−1^ followed by maintaining the samples at 300 °C for 5 min.

A flow rate of 2 mL min^−1^ was used to transport the carrier gas, which had a split ratio of 1:20. A mass selective detector, the Agilent 5975 C (Agilent Technologies, Palo Alto, CA, USA), was connected to a gas chromatograph. In addition to an ion-trap analyzer with a rate of 1508 amu s^−1^ and an electron multiplier voltage of 1350 V, the MS was equipped with a cyclotron. All of the MS data were collected as full-scan mass spectra in the range of 39–400 *m*/*z*. Finally, of all the essential oils, only the amounts of thymol and carvacrol were reported in this work, as they are the main essential oil compounds in thyme. This was confirmed by those reported in the literature [60].

### 3.8. Statistical Analysis

All experiments were analyzed as factorial based on completely randomized design (CRD) with three biological replications. The analysis of variance (ANOVA) for all of the data was performed using SPSS16 statistical software. Means comparison was performed using Duncan’s multiple range test (DMRT).

## 4. Conclusions

This study demonstrates the novel expression of three genes involved in the thymol/carvacrol biosynthetic pathway, for the first time. The total phenolic and flavonoid contents of Iranian thyme were determined in order to get a better knowledge of the thymol/carvacrol production in *T. vulgaris*, *T. migricus*, and *T. daenensis*. As a result of their pharmacological action, phenolic monoterpenes such as thymol and carvacrol have been identified as the primary active constituents of thyme essential oils. Thymol and carvacrol have also been identified as lead compounds. Genes involved in thymol/carvacrol production were differentially upregulated following treatment with MeJA. Our results showed that applying MeJA externally at different concentrations caused a significant increase in the amount of thymol, carvacrol, and total phenolic and flavonoid contents of thyme plants in three species compared to the control. The expression levels of TPS2, *CYP71D178*, and *CYP71D180* genes were dissimilar between different species of thymus depending on genotype. Thus, further investigation is needed to study the expression of other important genes involved in the thymol/carvacrol biosynthesis pathway as well as their relationship with thymol, carvacrol, and phenolic-compound accumulation under MeJa and other treatments with chemical elicitors. Our findings show that elicitation might help *T. vulgaris*, *T. migricus*, and *T. daenensis* produce more pharmaceutically active phenolic monoterpenes (thymol/carvacrol). In thymus and closely related plant species, an integrated study of metabolomics and transcriptomics might open the way for elucidating the regulatory mechanisms for fine tuning the production of pharmaceutically relevant chemicals.

## Figures and Tables

**Table 1:** Primers used to qRT-PCR analysis.

Real-Time Primers	Sequences (5′ to 3′)	Tm °C	Amplicon Size (bp)
TPS2 *F* TPS2 *R*	AACCTCGCCGAGAAACTCCC AGCTGCAGTTCGTCGAGTGT	62	182
*CYP71D178* *F* *CYP71D178* *R*	CGACCACTGGCGCCAAATAC CGATGGTGCACACCGTCTTG	60	179
*CYP71D180* *F* *CYP71D180* *R*	AACATCGGGTCGCGGATCAT GATGAGGCGAGCCATCTCGT	60	165
UQ *F* UQ *R*	AAGACCTACACCAAGCCCAA AAGTGAGCCCACACTTACCA	54	196

## Data Availability

Data is contained within this article.
